# Poly-*β*-(1→6)-*N*-acetyl-D-glucosamine mediates surface attachment, biofilm formation, and biocide resistance in *Cutibacterium acnes*

**DOI:** 10.3389/fmicb.2024.1386017

**Published:** 2024-05-01

**Authors:** Jeffrey B. Kaplan, Colette Cywes-Bentley, Gerald B. Pier, Nandadeva Yakandawala, Miloslav Sailer, Marc S. Edwards, Khalaf Kridin

**Affiliations:** ^1^Department of Biology, American University, Washington, DC, United States; ^2^Laboratory for Skin Research, Institute for Medical Research, Galilee Medical Center, Nahariya, Israel; ^3^Division of Infectious Diseases, Department of Medicine, Brigham and Women’s Hospital, Harvard Medical School, Boston, MA, United States; ^4^Kane Biotech Inc., Winnipeg, MB, Canada

**Keywords:** acne vulgaris, antibiofilm, antibiotic tolerance, benzoyl peroxide, biofilm matrix, dispersin B, DNase I, extracellular DNA

## Abstract

**Background:**

The commensal skin bacterium *Cutibacterium acnes* plays a role in the pathogenesis of acne vulgaris and also causes opportunistic infections of implanted medical devices due to its ability to form biofilms on biomaterial surfaces. Poly-*β*-(1→6)-*N*-acetyl-D-glucosamine (PNAG) is an extracellular polysaccharide that mediates biofilm formation and biocide resistance in a wide range of bacterial pathogens. The objective of this study was to determine whether *C. acnes* produces PNAG, and whether PNAG contributes to *C. acnes* biofilm formation and biocide resistance *in vitro*.

**Methods:**

PNAG was detected on the surface of *C. acnes* cells by fluorescence confocal microscopy using the antigen-specific human IgG1 monoclonal antibody F598. PNAG was detected in *C. acnes* biofilms by measuring the ability of the PNAG-specific glycosidase dispersin B to inhibit biofilm formation and sensitize biofilms to biocide killing.

**Results:**

Monoclonal antibody F598 bound to the surface of *C. acnes* cells. Dispersin B inhibited attachment of *C. acnes* cells to polystyrene rods, inhibited biofilm formation by *C. acnes* in glass and polypropylene tubes, and sensitized *C. acnes* biofilms to killing by benzoyl peroxide and tetracycline.

**Conclusion:**

*C. acnes* produces PNAG, and PNAG contributes to *C. acnes* biofilm formation and biocide resistance *in vitro*. PNAG may play a role in *C. acnes* skin colonization, biocide resistance, and virulence *in vivo*.

## Highlights

*Cutibacterium acnes* is a bacterium that is found on the skin of most people. *C. acnes* helps maintain a healthy skin microbiota but also causes acne and infections of implanted medical devices. In this study we found that *C. acnes* produces an adhesive extracellular polysaccharide named PNAG (poly-*N*-acetylglucosamine) which may help *C. acnes* colonize skin and medical implants. We found that PNAG protects *C. acnes* from killing by benzoyl peroxide and tetracycline, two drugs that are commonly used to treat acne. PNAG may represent a novel target for skin antiseptics and anti-acne drugs.

## Introduction

The anaerobic Gram-positive bacterium *Cutibacterium acnes* is an abundant colonizer of human skin (Achermann et al., 2014). Although considered a beneficial commensal, *C. acnes* can cause opportunistic invasive infections of the skin, soft tissue, cardiovascular system, and implanted medical devices (Coenye et al., 2022). *Cutibacterium acnes* also contributes the pathogenesis of the common inflammatory dermatosis acne vulgaris (McLaughlin et al., 2019).

Poly-*β*-(1→6)-*N*-acetyl-D-glucosamine (PNAG) is an extracellular polysaccharide that mediates biofilm formation, antimicrobial resistance, host colonization, immune evasion, and stress tolerance in a wide range of Gram-negative and Gram-positive bacterial pathogens (Cywes-Bentley et al., 2013; Soliman et al., 2018). The importance of PNAG as a bacterial colonization and virulence factor has been demonstrated in numerous animal studies using PNAG mutant strains (Kropec et al., 2005; Subashchandrabose et al., 2013; Chen et al., 2014; Shanmugam et al., 2015), anti-PNAG monoclonal antibodies (Cywes-Bentley et al., 2013), a PNAG vaccine (Gening et al., 2020), and the PNAG-specific glycoside hydrolase dispersin B (Serrera et al., 2007; Darouiche et al., 2009; Ragunath et al., 2011; Gawande et al., 2014; Kaplan et al., 2018).

Previous investigations of PNAG production in *C. acnes* were inconclusive. Okuda et al. (2018) detected *N*-acetylglucosamine (GlcNAc) in the extracellular biofilm matrix of five *C. acnes* strains isolated from infected cardiac pacemakers using a wheat germ agglutinin dot blot assay, but dispersin B did not inhibit biofilm formation by any of the five strains in 96-well polystyrene microplates, an indicator of PNAG production. Gannesen et al. (2019) observed no nuclear magnetic resonance (NMR) spectroscopic signal for PNAG in the biofilm matrix of *C. acnes* strain RT5, an acne isolate. In the present study, we reexamined PNAG production in *C. acnes* using an anti-PNAG monoclonal antibody and the PNAG-degrading enzyme dispersin B. Here we present evidence that *C. acnes* produces PNAG *in vitro*, and that PNAG mediates *C. acnes* surface attachment, biofilm formation, and resistance to killing by the anti-acne agents benzoyl peroxide and tetracycline.

## Materials and methods

### Bacterial strains and growth conditions

The bacterial strains used in this study are listed in Table 1. Strains were maintained on Tryptic Soy agar (BD). Bacterial inocula for broth cultures were prepared by transferring a loopful of cells from a 24-h-old agar plate to a microcentrifuge tube containing 200 μL of saline, mixing the cells by vortex agitation, and diluting the cells to 10^6^–10^7^ CFU/mL in filter-sterilized Tryptic Soy broth (BD). Culture vessels were 13 × 100 mm glass tubes or 15-mL conical-bottom polypropylene centrifuge tubes. Tubes were filled with 1-mL of inoculum and incubated anaerobically at 37°C for 72 h. Anaerobic conditions were created using a BD GasPak EZ Anaerobe sachet system.

**Table 1:** *Cutibacterium* spp. strains used in this work.

Strain	Source	Reference
*Cutibacterium* sp. KPL2009	Nostril isolate	Claesen et al. (2020)
*C. acnes* KPL1849	Nostril isolate	Wollenberg et al. (2014)
*C. acnes* HL036PA1	Acne lesion	Fitz-Gibbon et al. (2013)
*C. acnes* HL043PA1	Acne lesion	Fitz-Gibbon et al. (2013)
*C. acnes* HL072PA1	Acne lesion	Fitz-Gibbon et al. (2013)
*C. acnes* HL086PA1	Acne lesion	Fitz-Gibbon et al. (2013)

### Antimicrobial agents and enzymes

Tetracycline hydrochloride (Sigma-Aldrich; Catalog No. T7660) was dissolved at 10 mg/mL in distilled water, filter sterilized, and diluted in broth to the indicated concentrations. Benzoyl peroxide (TCI Chemicals; Catalog No. B3152) was dissolved at 10 mg/mL in dimethyl sulfoxide and diluted directly in broth. Sodium dodecyl sulfate (SDS; Catalog No. 428018) was purchased from Merck. Deoxyribonuclease I (Catalog No. DN25) was from Sigma-Aldrich. Dispersin B was obtained from Kane Biotech (Winnipeg MB, Canada).

### Fluorescence confocal microscopy

PNAG was detected on the surface of *Cutibacterium* spp. cells by fluorescence confocal microscopy using the PNAG-specific human IgG1 monoclonal antibody (mAb) F598 conjugated to Alexa Fluor 488 as previously described (Cywes-Bentley et al., 2013). Human IgG1 mAb F429, which binds to *Pseudomonas aeruginosa* alginate (Pier et al., 2004), was used as a negative control. Briefly, cells were swabbed from an agar plate onto glass microscope slides, air-dried, and covered for 1 min with ice-cold methanol. After rinsing, slides were reacted with 5.2 μg/mL mAb F598 or control mAb F429 directly conjugated to Alexa Fluor 488 along with 4 uM Syto 63 in BSA/PBS. After 2 h at room temperature, slides were washed and observed by confocal microscopy using a 63× oil objective.

### Crystal violet binding assay

Biofilms cultured in glass tubes were rinsed vigorously with tap water and stained for 1 min with 1 mL of Gram’s crystal violet. Tubes were then rinsed with tap water to remove the unbound dye, air-dried, and photographed. Tubes containing sterile broth were incubated and processed along with the inoculated tubes to serve as controls. To quantitate crystal violet binding, stained tubes were filled with 1 mL of 33% acetic acid, incubated at room temperature for 30 min, and mixed by vortex agitation. A volume of 200 μL of the dissolved dye was transferred to the well of a 96-well microtiter plate and its absorbance at 595 nm (*A*595) was measured in a microplate reader. Biofilm inhibition by dispersin B was calculated using the formula 1 − (*A*595_Dispersin B_/*A*595_No enzyme_) × 100.

### Surface attachment assay

*Cutibacterium acnes* cells were scraped from an agar plate and resuspended in PBS at *ca.* 10^6^ CFU/mL. Cell suspensions were filtered through a 5-μm pore-size syringe filter to remove large clumps of cells and then aliquoted into three 15-mL centrifuge tubes (2.5 mL/tube). The first tube was left untreated to serve as a control. The second tube was supplemented with 20 μg/mL of dispersin B. The third tube was supplemented with 20 μg/mL of heat-inactivated dispersin B (95°C, 10 min). After 30 min at 37°C, the tubes were mixed by vortex agitation, and four 0.5-mL aliquots of each cell suspension were transferred to four separate 1.5-mL microcentrifuge tubes (0.5 mL/tube). A 25-mm long ethanol-sterilized polystyrene rod (1.5 mm diam; Plastruct Inc., Des Plaines IL, United States) was placed in each microcentrifuge tube. After 30 min, the rods were removed, rinsed with PBS to remove loosely adherent cells, and transferred to 15-mL conical centrifuge tubes containing 1 mL of PBS. Cells were detached from the rods by sonication, diluted, and plated on agar for CFU enumeration.

### Autoaggregation assay

*Cutibacterium acnes* cells were scraped from an agar plate into 2 mL of PBS using a cell scraper. Aliquots of the cell suspension were treated with 20 μg/mL dispersin B or 10 μg/mL DNase I for 15 min. One aliquot of cells was left untreated to serve as a control. A total of 300 μL of each cell suspension was transferred to a 0.5-mL polypropylene centrifuge tube (model 6,530; Corning). The tube was then mixed by high-speed vortex agitation for 10 s, incubated statically for 20 min, and photographed.

### Treatment of biofilms with enzymes and detergent

72-h-old biofilms grown in glass tubes were rinsed vigorously with water and then treated with 1 mL of 20 μg/mL dispersin B, 10 μg/mL DNase I, or 1% SDS. After 15 min, tubes were rinsed vigorously with water and stained with crystal violet as described above.

### Benzoyl peroxide killing assay

72-h-old biofilms grown in glass tubes were treated directly with 20 μg/mL dispersin B in PBS for 15 min followed by 70 or 140 μg/mL benzoyl peroxide for 10 min. Biofilms were then rinsed, detached from the tubes by sonication, diluted, and plated on agar for CFU enumeration.

### Tetracycline tolerance assay

Biofilms were cultured in glass tubes in broth supplemented with 100 μg/mL dispersin B and/or 0.2 μg/mL tetracycline (MIC = 0.5 μg/mL). After 72 h, biofilms were rinsed, detached from the tubes by sonication, diluted, and plated on agar for CFU enumeration.

### Statistics and reproducibility of results

All experiments were performed in triplicate or quadruplicate tubes. All experiments were performed 2–3 times with similar results. The significance of differences between means was calculated using a Student’s *t*-test. A *p*-value < 0.01 was considered significant.

## Results

### Detection of PNAG on *Cutibacterium* spp. cells

**Figure 1:**
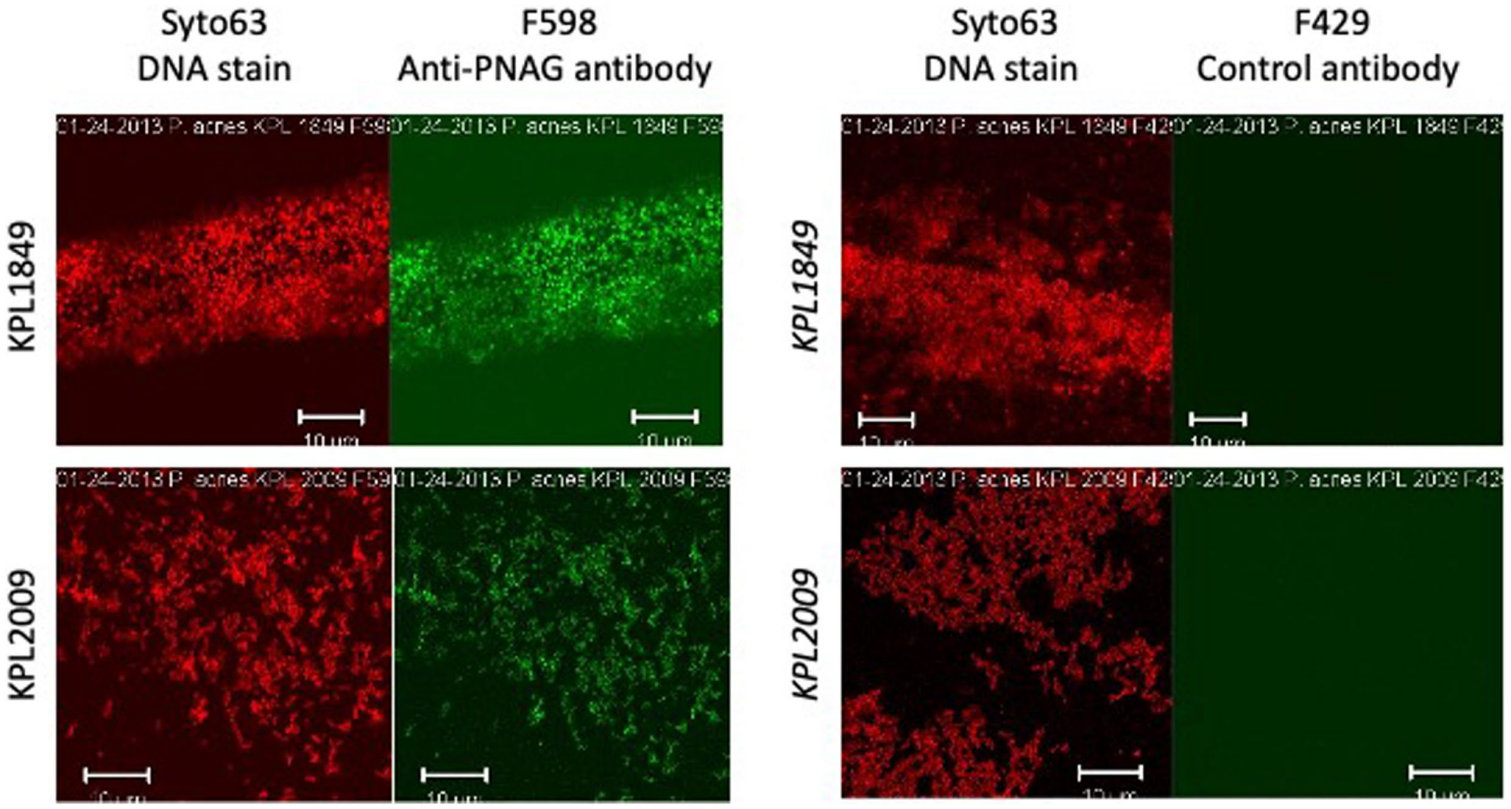
Fluorescence confocal microscopic images of *Cutibacterium acnes* strain KPL1849 (top) and *Cutibacterium* sp. strain KPL2009 (bottom) stained with Syto 63 and Alexa Fluor 488-conjugated anti-PNAG mAb F598 (left panels) or Syto 63 and Alexa Fluor 488-conjugated control antibody F429 which binds to *Pseudomonas aeruginosa* alginate (right panels). Syto 63 stains DNA red and mAb F598 stains PNAG green. Stains are indicated above. Strain names are indicated at the left. Measure bars = 10 μm.

Cells of *Cutibacterium* sp. strain KPL2009 and *C. acnes* strain KPL1849 were reacted with PNAG-specific mAb F598 or control mAb F429, both directly conjugated to Alexa Fluor 488, and then visualized for immunofluorescence by confocal microscopy (Figure 1). Bacteria embedded in an immunoreactive matrix of PNAG were observed with mAb F598 but not with control mAb F429, suggesting that both strains produce PNAG.

### Dispersin B inhibits *Cutibacterium acnes* biofilm formation in glass tubes

**Figure 2:**
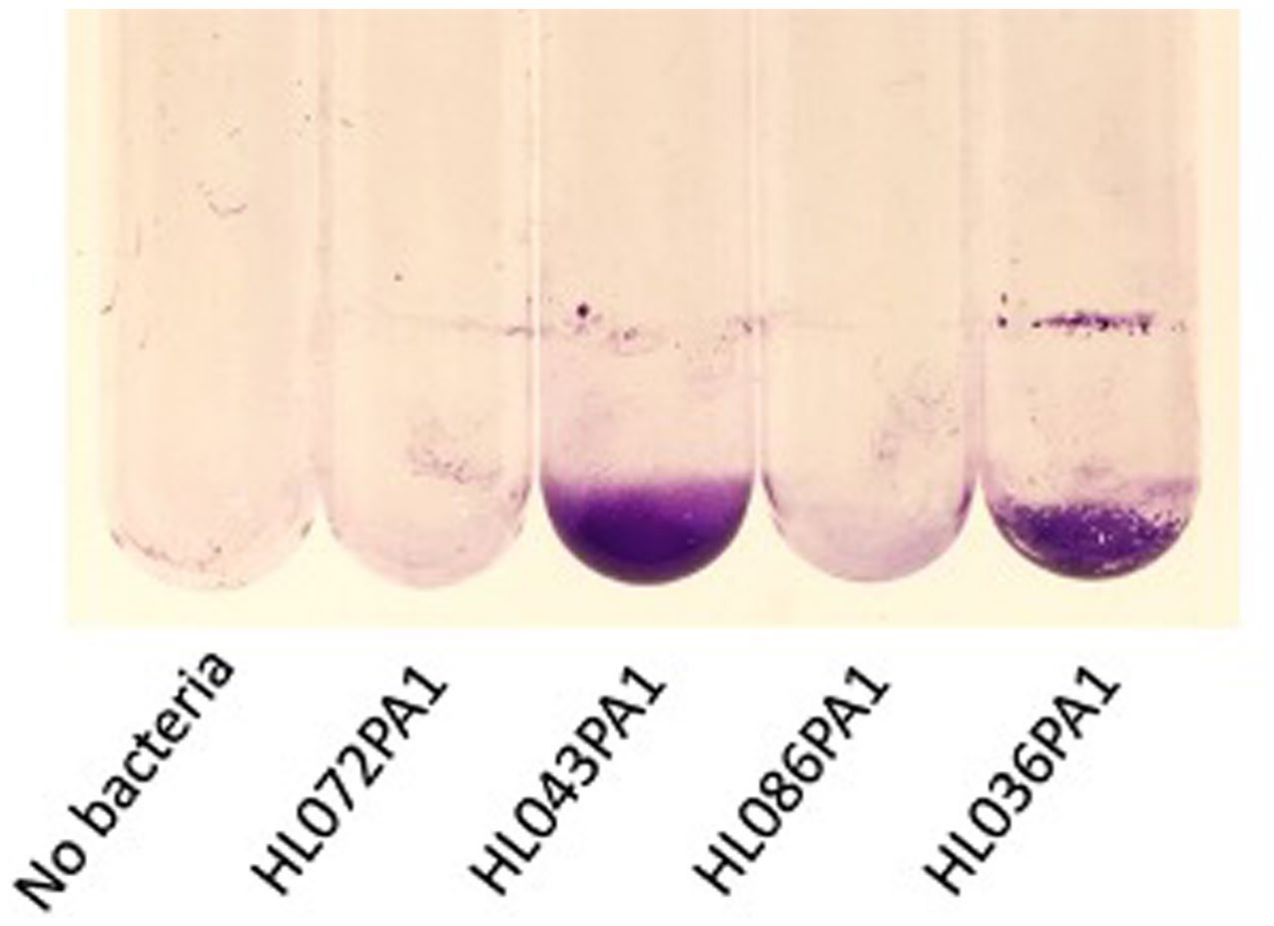
Biofilm formation by four *Cutibacterium acnes* strains in 13 × 100 mm glass tubes. Cultures were incubated for 3 days, rinsed with water, and stained with crystal violet. Strain names are indicated below. The tube at the left was incubated with sterile broth. This experiment was performed in duplicate tubes on three separate occasions with identical results. Representative tubes are shown.

**Figure 3:**
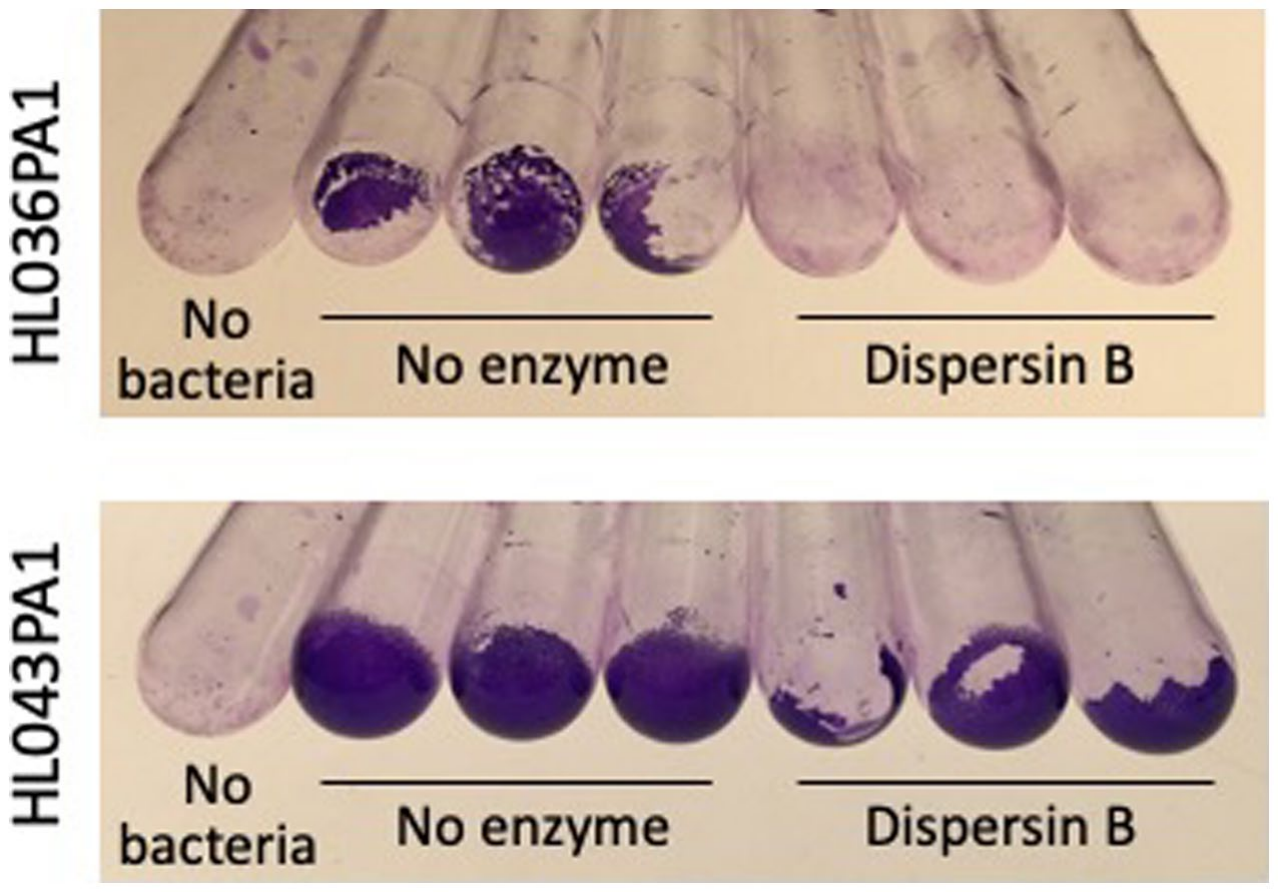
Biofilm formation by *Cutibacterium acnes* strains HL036PA1 and HL043PA1 in the absence or presence of 100 μg/mL dispersin B. Biofilms were cultured in glass tubes for 3 days, then rinsed with water and stained with crystal violet. Triplicate tubes for each condition are shown. The control tubes at the left (No bacteria) were incubated with sterile broth. These experiments were performed on two separate occasions with similar results. Tubes from representative experiments are shown.

The ability of four *C. acnes* strains to form biofilms in glass culture tubes was investigated using a crystal violet binding assay (Figure 2). Two of the four strains tested (HL043PA1 and HL036PA1) formed strong biofilms as evidenced by the large amount of bound crystal violet dye at the bottom of the tube. To determine whether dispersin B inhibits *C. acnes* biofilm formation, biofilm-forming strains HL043PA1 and HL036PA1 were incubated in unsupplemented broth or broth supplemented with 100 μg/mL dispersin B (Figure 3). Dispersin B significantly inhibited biofilm formation by both strains as evidenced by a lower amount of bound crystal violet dye in tubes supplemented with the enzyme. Quantitation of bound dye for strain HL034PA1 yielded a biofilm inhibition value of 99% compared to the no enzyme control (*p* < 0.001).

### Dispersin B inhibits attachment of *Cutibacterium acnes* cells to polystyrene rods

**Figure 4:**
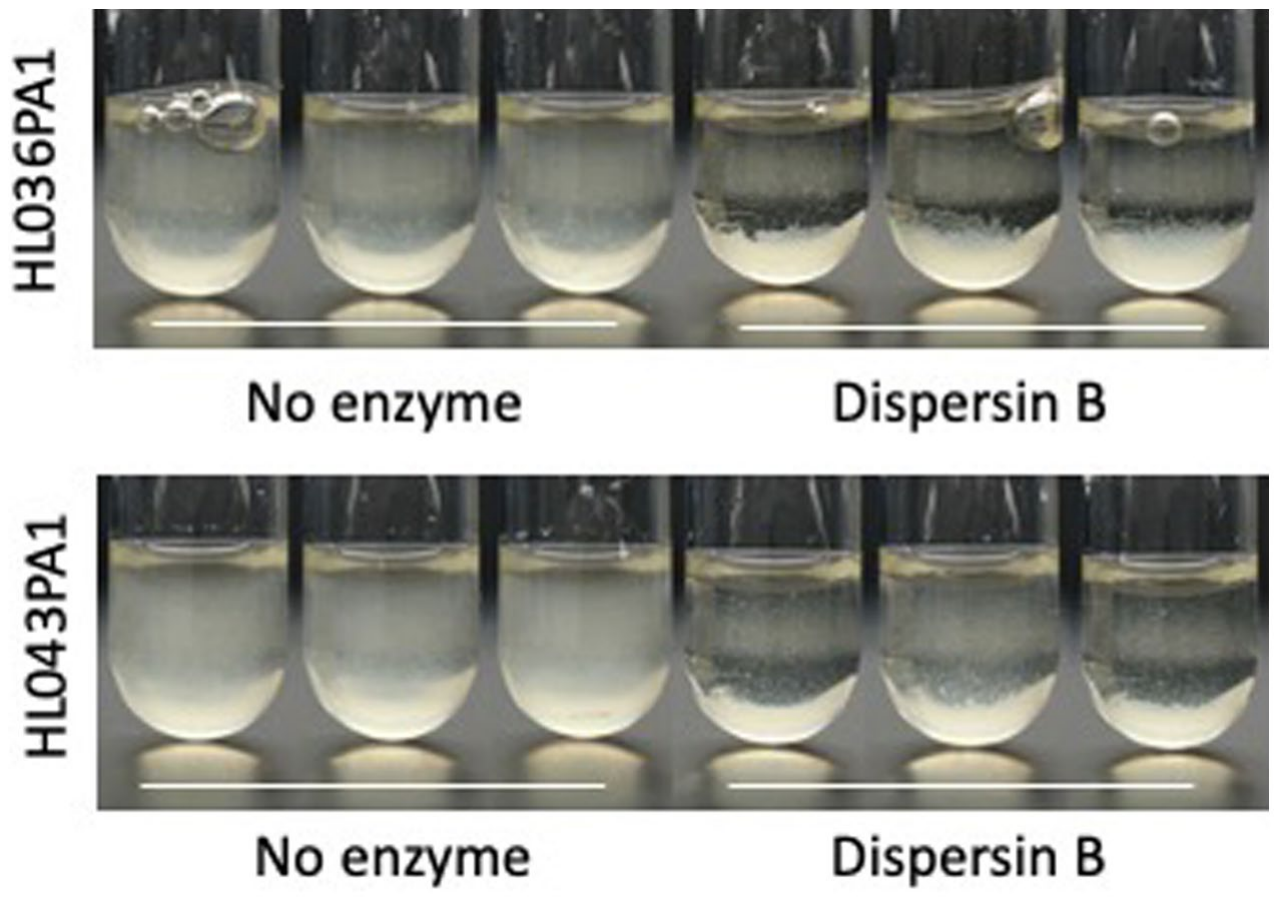
Growth of *Cutibacterium acnes* strains HL036PA1 and HL043PA1 in glass tubes in the presence of 0 or 100 μg/mL dispersin B. Tubes were photographed after 3 days of incubation. Triplicate tubes for each condition are shown. Strain names are indicated at the left. Enzyme treatments are indicated below. These experiments were performed on three separate occasions with identical results. Tubes from representative experiments are shown.

**Figure 5:**
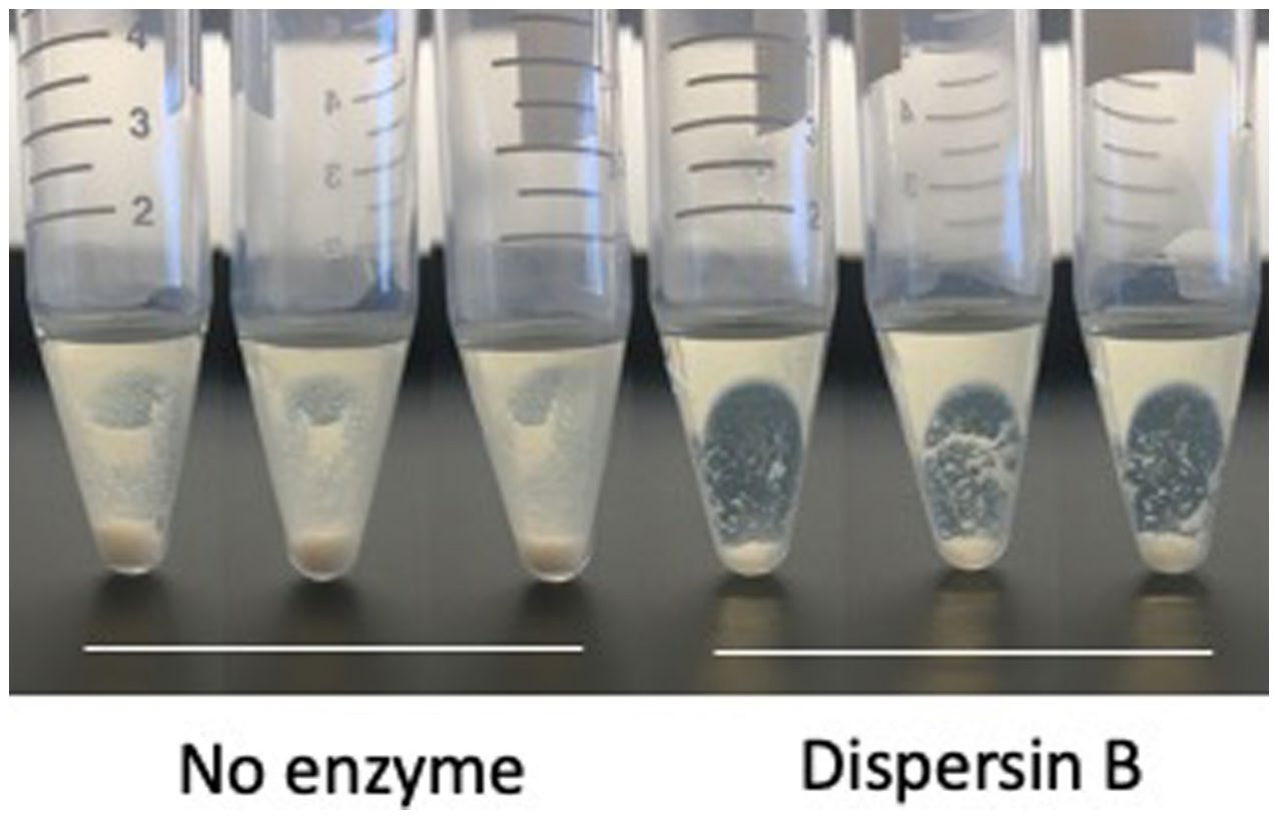
Growth of *Cutibacterium acnes* strain HL036PA1 in polypropylene tubes in the presence of 0 or 100 μg/mL dispersin B. Bacteria were photographed after 3 days of growth. Triplicate tubes for each condition are shown. This experiment was performed on two separate occasions with identical results. Tube from one representative experiment are shown.

**Figure 6:**
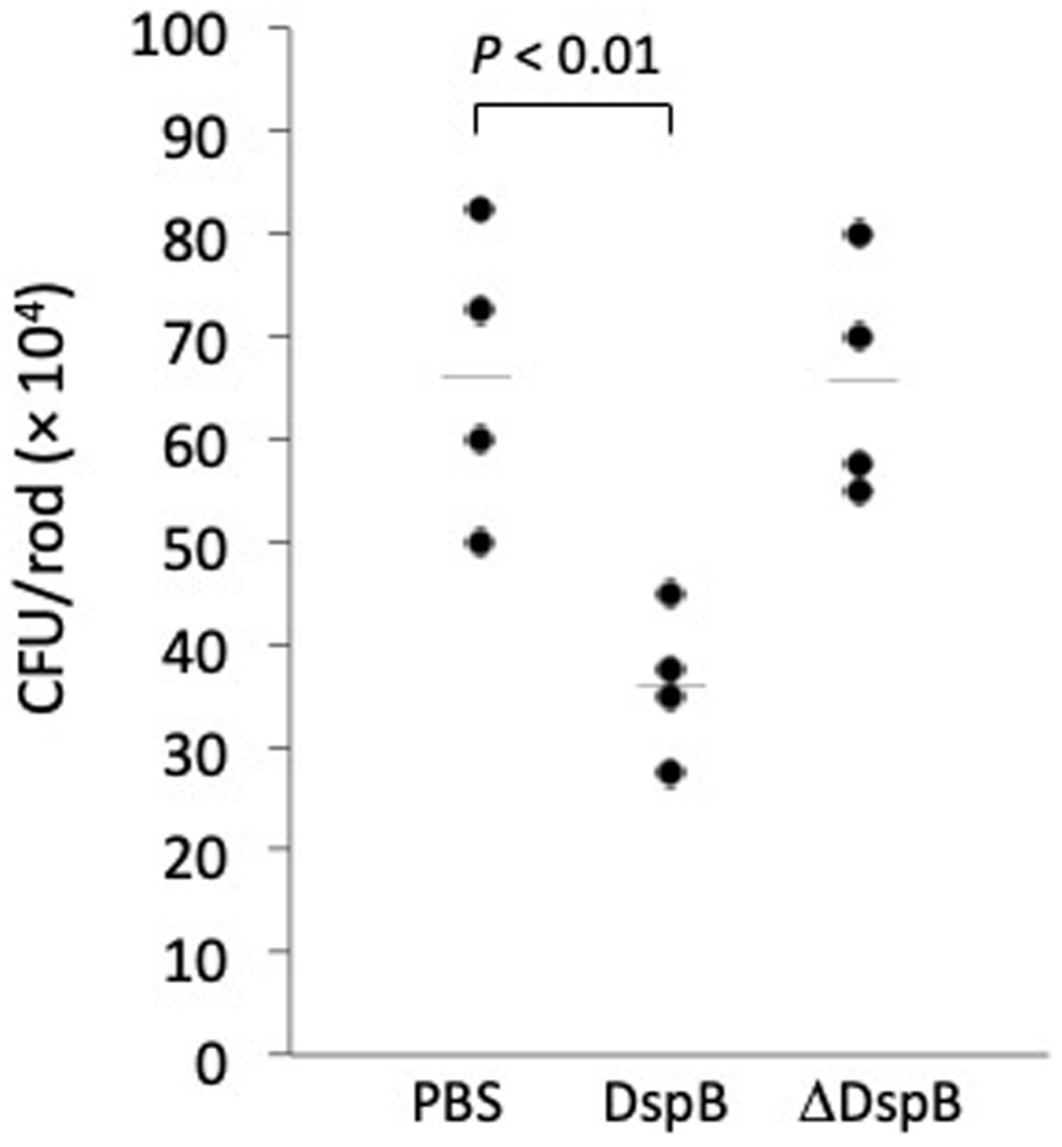
Attachment of *Cutibacterium acnes* HL043PA1 planktonic cells to polystyrene rods. Cells were treated with phosphate buffered saline (PBS), 20 μg/mL dispersin B (DspB), or 20 μg/mL heat-inactivated dispersin B (ΔDspB) for 15 min prior to contacting the rods. Each point represents one individual rod. Horizontal lines indicate mean values. This experiment was performed on two separate occasions with similar results. Results from one representative experiment are shown.

Photographs of HL043PA1 and HL036PA1 glass culture tubes taken directly after incubation (prior to rinsing and crystal violet staining) revealed the presence of thin biofilms along the sides of the tubes that were absent in tubes supplemented with dispersin B (Figure 4). This phenomenon was also evident for HL043PA1 and HL036PA1 cultured in conical-bottom polypropylene centrifuge tubes (Figure 5 and data not shown). In both types of tubes, however, a significant amount of cell clumping was observed at the bottom of the tube, even in the presence of the enzyme. These observations suggest that PNAG may promote the attachment of *C. acnes* cells and biofilms to surfaces. To test this hypothesis, untreated and dispersin B-treated *C. acnes* planktonic cells were incubated in the presence of polystyrene rods and the number of cells that attached to the rods after 30 min was enumerated (Figure 6). Significantly fewer dispersin B-treated cells attached to the rods than untreated cells, whereas cells treated with heat-inactivated dispersin B attached to the rods at the same level as untreated cells. These results suggest that PNAG contributes to *C. acnes* surface attachment.

### DNase I inhibits *Cutibacterium acnes* autoaggregation

**Figure 7:**
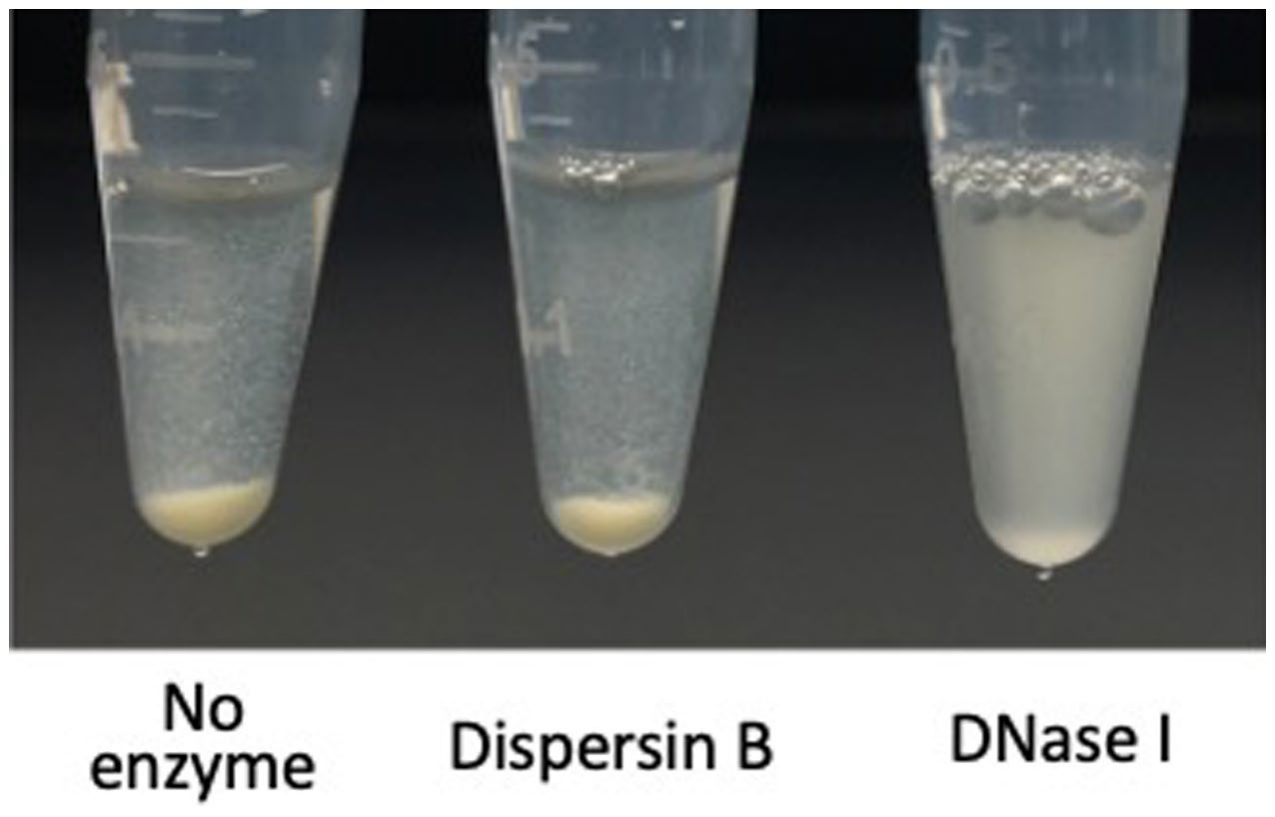
Autoaggregation of *Cutibacterium acnes* HL043PA1 cells in the presence of 20 μg/mL dispersin B or 10 μg/mL DNase I. Cell suspensions supplemented with the indicated enzyme were transferred to polypropylene tubes, incubated statically for 20 min, and then photographed. This experiment was performed in duplicate tubes on two separate occasions with identical results. Representative tubes are shown.

Autoaggregation (also termed intercellular adhesion) often plays a role in biofilm formation (Trunk et al., 2018). To test whether PNAG contributes to *C. acnes* autoaggregation, *C. acnes* HL036PA1 cells were treated with dispersin B or DNase I for 30 min, mixed by vortex agitation, transferred to a microcentrifuge tube, allowed to settle for 15 min, then photographed (Figure 7). Untreated control cells and dispersin B-treated cells settled to the bottom of the tube, whereas DNase I-treated cells remained in suspension. These findings suggest that extracellular DNA, but not PNAG, contributes to *C. acnes* autoaggregation.

### DNase I and SDS detach pre-formed *Cutibacterium acnes* biofilms from glass tubes

**Figure 8:**
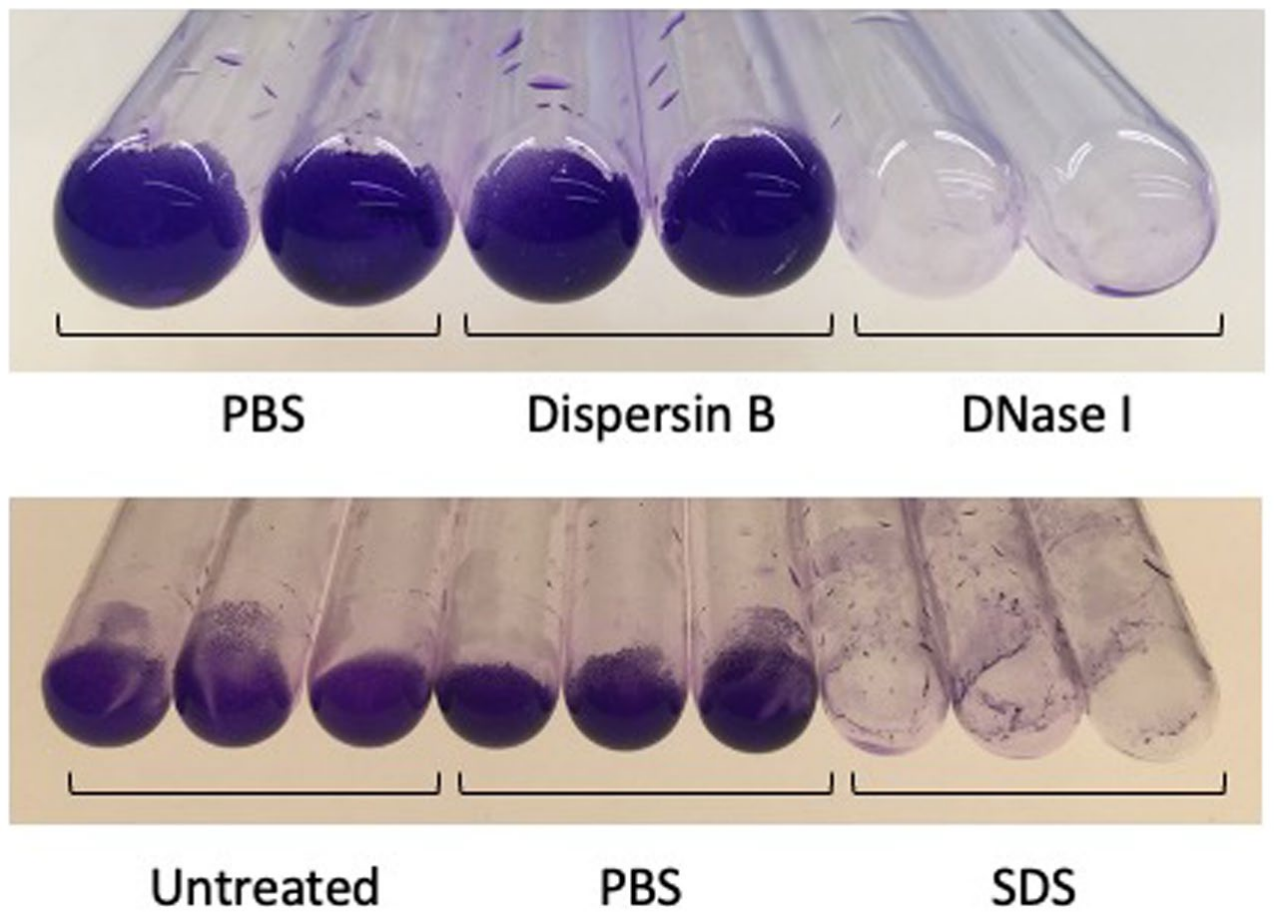
Detachment of 3-days-old *Cutibacterium acnes* HL043PA1 biofilms by enzymes and detergents. Biofilms were rinsed with water, treated with the indicated agent for 30 min at 37°C, re-rinsed, and stained with crystal violet. Dispersin B was 50 μg/mL, DNase I was 10 μg/mL, and sodium diodecyl sulfate (SDS) was at 1%. Duplicate tubes for each treatment are shown in the top panel and triplicate tubes for each treatment are shown in the bottom panel. These experiments were performed on three separate occasions with identical results. Representative experiments are shown.

To further investigate the composition of *C. acnes* biofilms, 72-h-old HL043PA1 biofilms were treated with dispersin B, DNase I, or SDS for 15 min, and then stained with crystal violet to visualize the biofilm remaining after treatment (Figure 8). DNase I and SDS, but not dispersin B, efficiently detached the mature biofilms, suggesting that extracellular DNA and proteinaceous adhesins, but not PNAG, contribute to biofilm stability in mature *C. acnes* biofilms.

### Dispersin B sensitizes *Cutibacterium acnes* biofilms to benzoyl peroxide killing

**Figure 9:**
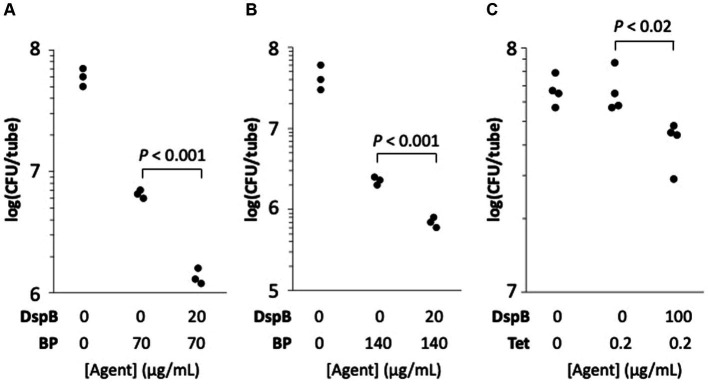
Dispersin B (DspB) sensitizes *Cutibacterium acnes* HL043PA1 biofilms to killing by benzoyl peroxide (BP) **(A,B)** and to growth inhibition by tetracycline (Tet) **(C)**. Panels **(A,B)** show the effect of a 10-min BP treatment on 72-h-old biofilms cultured in glass tubes. Panel **(A)** 70 μg/mL BP; panel **(B)**, 140 μg/mL BP. Some biofilms were treated with 20 μg/mL DspB in PBS for 15 min prior to contact with BP as indicated in the legends below. In panel **(C)**, biofilms were cultured for 72-h in 0 or 0.2 μg/mL Tet. Some tubes were supplemented with 100 μg/mL DspB as indicated in the legend below the graph. Each dot represents one individual tube. The experiment in panel **(C)** was performed on two separate occasions with similarly significant differences between tetracycline alone and tetracycline + dispersin B.

HL043PA1 biofilms (72-h-old) were treated directly with 20 μg/mL dispersin B for 15 min followed by 70 or 140 μg/mL benzoyl peroxide for 10 min. The biofilms were then detached from the tubes by sonication, diluted, and plated on agar for CFU enumeration. Control experiments showed that dispersin B alone did not kill *C. acnes* cells or inhibit their growth (data not shown). Treatment of *C. acnes* biofilms with 70 or 140 μg/ mL benzoyl peroxide alone resulted in a 1-log reduction in *C. acnes* CFUs, while pre-treatment of biofilms with dispersin B increased benzoyl peroxide killing by approximately 0.5 log (*p* < 0.001; Figures 9A,B). These findings suggest that PNAG protects *C. acnes* biofilm cells from killing by benzoyl peroxide.

### Dispersin B decreases tetracycline tolerance in *Cutibacterium acnes* biofilms

The effect of dispersin B on the tolerance of HL043PA1 biofilms to tetracycline was measured by culturing biofilms in broth supplemented with 100 μg/mL dispersin B and/or 0.2 μg/mL tetracycline (MIC = 0.5 μg/mL). After 72 h, fewer *C. acnes* cells were recovered from tubes supplemented with dispersin B plus tetracycline compared to tubes supplemented with tetracycline alone (*p* < 0.02; Figure 9C). These findings suggest that PNAG contributes to tetracycline tolerance *C. acnes* biofilms.

## Discussion

*Cutibacterium acnes* is one of the most abundant bacteria on the skin of most people (Oh et al., 2014). *Cutibacterium acnes* is both a beneficial commensal that helps maintain homeostasis of the skin microbiome, and an opportunistic pathogen associated with acne vulgaris and invasive infections of implanted medical devices (Achermann et al., 2014). Biofilm formation likely plays an important role in the ability of *C. acnes* to colonize skin and device surfaces (Coenye et al., 2022). Biofilms may also play a role in the pathogenesis of acne vulgaris (McLaughlin et al., 2019). *Cutibacterium acnes* biofilms have been observed in acne lesions and on implanted medical devices *in vivo* (Bayston et al., 2006; Jahns et al., 2012). Biofilm formation is also a common phenotype among *C. acnes* clinical isolates *in vitro* (Coenye et al., 2022). Understanding the mechanism of *C. acnes* biofilm formation may lead to the development of novel antibiofilm agents that can be used to treat acne or prevent invasive infections.

Bacteria in a biofilm are encased in a self-synthesized polymeric matrix that holds the cells together in a mass, attaches them to a tissue or surface, and protects them from killing by biocides and host immunity (Flemming and Wingender, 2010). Several previous studies have investigated the composition of the *C. acnes* biofilm matrix *in vitro*. Gannesen et al. (2019) found that the biofilm matrix of *C. acnes* acneic strain RT5 consisted of approximately 63% polysaccharides, 10% proteins, 4% DNA, and 23% other compounds. The major polysaccharide was a linear polymer of glucose, galactose, mannose, galactosamine, and diaminomannuronic acid in a molar ratio of 1:1:0.3:1:2. Jahns et al. (2016) detected similar components in the biofilm matrix of *C. acnes* skin isolate KPA171202 by performing fluorescent microscopy with carbohydrate-, protein-, and DNA-specific stains. Kuehnast et al. (2018) found that proteinase K significantly detached pre-formed biofilms produced by 7 of 8 *C. acnes* strains in flow-cells, and that DNase I detached 4 of the 8 strains tested. Similarly, Fang et al. (2021) found that cationic liposomes loaded with DNase I or proteinase K significantly detached pre-formed biofilms produced by *C. acnes* acneic strain ATCC6919 in 24-well microtiter plate wells, and Okuda et al. (2018) found that proteinase K and DNase I significantly inhibited biofilm formation by 2 of 5 *C. acnes* implant isolates in 96-well microplates when the enzymes were added to the culture medium prior to biofilm formation. Taken together, these results are consistent with the presence of polysaccharides, proteinaceous adhesins and eDNA in the biofilm matrix of some *C. acnes* strains. Our results demonstrating inhibition of HL043PA1 autoaggregation by DNase I (Figure 7) and detachment of *C. acnes* HL043PA1 biofilms by DNase I and SDS (Figure 8) are consistent with the presence of proteinaceous adhesins and eDNA in the biofilm matrix of this strain. Several studies have revealed an important role for eDNA in biofilm formation, adhesion, and structural integrity in diverse bacterial species (Panlilio and Rice, 2021).

Previous investigations of PNAG production in *C. acnes* were inconclusive. Gannesen et al. (2019) observed no NMR spectroscopic signal for *N*-acetylglucosamine, 2-acetamido-2-deoxy-galactose, or PNAG in the biofilm matrix of *C. acnes* strain RT5, suggesting that this strain does not produce PNAG. Okuda et al. (2018) found that dispersin B did not inhibit biofilm formation by five strains of *C. acnes* isolated from cardiac pacemakers when cultured in polystyrene microtiter plate wells. Since dispersin B was previously shown to inhibit biofilm formation by other PNAG-producing bacteria *in vitro* (Itoh et al., 2005; Parise et al., 2007; Pérez-Mendoza et al., 2011; Turk et al., 2013), these results suggested that PNAG was not a major adhesive component of biofilms produced by these five *C. acnes* strains.

In the present study we reinvestigated PNAG production in *C. acnes* using the PNAG-specific mAb F598 and the PNAG-specific glycosidase dispersin B. We found that mAb F598 reacted with cells of *C. acnes* strain KPL1849 and *Cutibacterium* sp. strain KPL2009 cultured on agar (Figure 1), suggesting that these two strains produce PNAG under some conditions. We also found that dispersin B exhibited antibiofilm activities against *C. acnes* strains HL036PA1 and HL043PA1 including inhibition of surface attachment (Figures 3–6) and sensitization to biocide killing (Figure 9). Since dispersin B is an accurate indicator of PNAG production (Cywes-Bentley et al., 2013; Eddenden and Nitz, 2022), these findings suggest that these two *C. acnes* strains also produce PNAG under some conditions. The antibiofilm activities exhibited by dispersin B against *C acnes* HL036PA1 and HL043PA1 are consistent with those exhibited by dispersin B against other species of bacteria (Itoh et al., 2005; Izano et al., 2007; Parise et al., 2007; Ganeshnarayan et al., 2009). The lack of a NMR signal for PNAG in the biofilm matrix of *C. acnes* strain RT5 (Gannesen et al., 2019) may be due to the fact that not all *C. acnes* strains produce PNAG, or that PNAG is a minor component of the RT5 biofilm matrix. The fact that dispersin B did not exhibit biofilm inhibiting activity against five implant-associated C. acnes strains in 96-well microplates (Okuda et al., 2018) may be due to strain differences or to the fact that dispersin B exhibits different antibiofilm activities depending on the shape and size of the culture vessel (Izano et al., 2007). It is also possible that the enzyme used by Okuda et al. (2018) was inactive because no positive control for enzyme activity was reported.

Our results suggest that PNAG mediates *C. acnes* surface attachment and biocide resistance, but that eDNA is the major intercellular adhesin in mature *C. acnes* biofilms. *C. acnes* be similar to *Staphylococcus aureus*, where double-stranded DNA is the most common extracellular component of biofilms produced by most strains (Sugimoto et al., 2018), while PNAG functions to confer resistance to killing by biocides (Serrera et al., 2007; Darouiche et al., 2009; Gawande et al., 2014) and innate host immune mediators (Kropec et al., 2005). Like *C. acnes*, *S. aureus* biofilms are readily detached by DNase I but not by dispersin B (Izano et al., 2008; Kaplan et al., 2012). More experiments are needed to determine the functions of PNAG in *C. acnes* cells and biofilms, and to determine whether PNAG production correlates with skin colonization, biocide resistance, immune tolerance, acne pathogenesis, and medical device infections *in vivo*.

Previous *in vivo* studies showed that PNAG is an important colonization and virulence factor for numerous other bacterial pathogens including *Aggregatibacter actinomycetemcomitans* (Shanmugam et al., 2015); *Klebsiella pneumoniae* (Chen et al., 2014); *Staphylococcus epidermidis* (Kaplan et al., 2018), *Staphylococcus aureus* (Kropec et al., 2005; Serrera et al., 2007; Darouiche et al., 2009; Gawande et al., 2014), *Pectobacterium carotovorum* (Ragunath et al., 2011) and *Actinobacillus pleuropneumoniae* (Subashchandrabose et al., 2013). PNAG may similarly contribute to *C. acnes* surface attachment, biofilm formation, biocide tolerance, skin colonization, acne pathogenesis and medical device infections *in vivo*. PNAG may enable *C. acnes* to form biofilms and colonize epithelial surfaces and hair follicles, to form biofilms on biomaterials, and to resist killing by antimicrobial agents and host immunity. It is also possible that PNAG functions as a biological glue that holds corneocytes together to form acne microcomedones (Burkhart and Burkhart, 2007).

## Data availability statement

The original contributions presented in the study are included in the article/supplementary material, further inquiries can be directed to the corresponding author.

## Author contributions

JK: Conceptualization, Data curation, Formal analysis, Funding acquisition, Investigation, Methodology, Project administration, Resources, Software, Supervision, Validation, Visualization, Writing – original draft, Writing – review & editing. CC-B: Conceptualization, Data curation, Formal analysis, Investigation, Methodology, Project administration, Resources, Software, Supervision, Validation, Visualization, Writing – original draft, Writing – review & editing. GP: Conceptualization, Data curation, Funding acquisition, Resources, Supervision, Writing – original draft, Writing – review & editing. NY: Writing – review & editing. MS: Writing – review & editing. ME: Funding acquisition, Writing – review & editing. KK: Funding acquisition, Writing – review & editing.
